# Improving the healthcare response to domestic violence and abuse in sexual health clinics: feasibility study of a training, support and referral intervention

**DOI:** 10.1136/sextrans-2016-052866

**Published:** 2017-07-19

**Authors:** Alex Hardip Sohal, Neha Pathak, Sarah Blake, Vanessa Apea, Judith Berry, Jayne Bailey, Chris Griffiths, Gene Feder

**Affiliations:** 1 Queen Mary University of London, Centre for Primary Care and Public Health, Barts and The London School of Medicine and Dentistry, London, UK; 2 Women’s Health Research Unit, Queen Mary University of London, University of Bristol School of Social and Community Medicine, Bristol, UK; 3 University of Bristol, School of Social and Community Medicine, Bristol, UK; 4 Barts Health NHS Trust, Sexual Health, London, UK; 5 University Hospitals Bristol NHS Foundation Trust, Bristol Sexual Health Centre, Bristol, UK; 6 University of Bristol, School of Social and Community Medicine, Bristol, UK; 7 Queen Mary University of London, Centre for Primary Care and Public Health, London, UK; 8 University of Bristol, Centre for Academic Primary Care, Bristol, UK

**Keywords:** Complex interventions, Evidence based medicine, Public health, Women, Health serv research

## Abstract

**Objectives:**

Sexual health and gynaecological problems are the most consistent and largest physical health differences between abused and non-abused female populations. Sexual health services are well placed to identify and support patients experiencing domestic violence and abuse (DVA). Most sexual health professionals have had minimal DVA training despite English National Institute for Health and Care Excellence recommendations. We sought to determine the feasibility of an evidence-based complex DVA training intervention in female sexual health walk-in services (IRIS ADViSE: Identification and Referral to Improve Safety whilst Assessing Domestic Violence in Sexual Health Environments).

**Methods:**

An adaptive mixed method pilot study in the female walk-in service of two sexual health clinics. Following implementation and evaluation at site 1, the intervention was refined before implementation at site 2. The intervention comprised electronic prompts, multidisciplinary training sessions, clinic materials and simple referral pathways to IRIS ADViSE advocate-educators (AEs). The pilot lasted 7 weeks at site 1 and 12 weeks at site 2. Feasibility outcomes were to assign a supportive DVA clinical lead, an IRIS ADViSE AE employed by a local DVA service provider, adapt electronic records, develop local referral pathways, assess whether enquiry, identification and referral rates were measurable.

**Results:**

Both sites achieved all feasibility outcomes: appointing a supportive DVA clinical lead and IRIS ADViSE AE, establishing links with a local DVA provider, adapting electronic records, developing local referral pathways and rates of enquiry, identification and referral were found to be measurable. Site 1: 10% enquiry rate (n=267), 4% identification rate (n=16) and eight AE referrals. Site 2: 61% enquiry rate (n=1090), a 7% identification rate (n=79) and eight AE referrals.

**Conclusions:**

IRIS ADViSE can be successfully developed and implemented in sexual health clinics. It fulfils the unmet need for DVA training. Longer-term evaluation is recommended.

## Introduction

Domestic violence and abuse (DVA) is a major public health and clinical problem affecting individuals, families, communities and society. The UK intergovernmental definition of DVA is any incident or pattern of controlling, coercive or threatening behaviour, violence or abuse between people aged ≥16 years who are or have been intimate partners or family members, regardless of gender or sexuality.^1^ DVA can encompass, but is not limited to, psychological, physical, sexual, financial or emotional abuse. The estimated annual cost of DVA to the British National Health Service is £1.7 billion per year, additional mental health costs £176 million^2^ and the aggregate UK cost including lost economic output, social services, medical and emotional costs an estimated £11 billion.^3^ Although DVA affects men and women, the prevalence of all DVA is higher among women and increasing since 2008/2009.^4^ Women experience more severe and repeated physical abuse, much more sexual abuse and more coercive control than men.^1^

Gynaecological and sexual health problems are the most prevalent and persistent physical health consequence of DVA. Presentations include STIs, painful sex, chronic pelvic pain, vaginal bleeding and recurrent urinary tract infections. Risk of these problems is threefold higher in abused women, and increases in a dose–response fashion with coexisting sexual and physical abuse.^5^ DVA is also associated with an increased risk of unintended pregnancy, induced abortion, increased sexual risk taking and inconsistent condom use.^6 7^ Lifetime prevalence of DVA in women attending sexual health services (47%) is higher than in the general population.^8^

Sexual health services can be the first point of contact for women who have experienced DVA, so is an appropriate setting in which to identify and support women affected by DVA.^9^ They were listed as a setting in which all patients should be asked about DVA as a ‘routine part of good clinical practice’ by the National Institute for Health and Care Excellence,^10^ which develops evidence-based guidance for healthcare in England. However, most sexual health professionals have had minimal training in identifying and responding to DVA.

IRIS (Identification and Referral to Improve Safety) is an evidence-based complex intervention including DVA training, support and referrals to specialist DVA advocate-educators (AE) in primary care, designed to improve the healthcare response to women affected by DVA. A cluster randomised controlled trial in Hackney and Bristol general practices showed that IRIS increased the identification of women affected by DVA (currently or historically) 3-fold, discussion about referral 22-fold and actual referral 6-fold.^11^ The IRIS intervention is likely to be cost-effective^12^ as well as acceptable to service users^13^ and professionals.^14^ The IRIS model has been endorsed by the Royal College of General Practitioners and is frequently cited in guidance as good practice.^10 15–17^ IRIS has been commissioned in 34 localities, involving >1000 UK general practices.

### Aim

Determining the feasibility of developing and implementing an IRIS-type model in sexual health services: IRIS ADViSE (Assessing Domestic Violence in Sexual Health Environments).

## Methods

### Design

We used an adaptive pilot study design^18^ where the experience and data at site 1, in East London, led to modifications at site 2, in Bristol. We used a mixed methods approach to evaluation. This comprised monitoring of predefined feasibility outcomes which included analysis of numerical data extracted from the electronic medical record. Qualitative analysis of staff interviews is reported in a separate paper.^19^

### Setting and participants

Both sites were female walk-in sexual health services. Site 1 was an east London clinic, serving an inner-city multiethnic population. Site 2 was a Bristol clinic, serving an urban population. Both clinics saw commuters and those referred from primary care, community and regional clinics.

### Process, intervention and procedures

**Figure 1:**
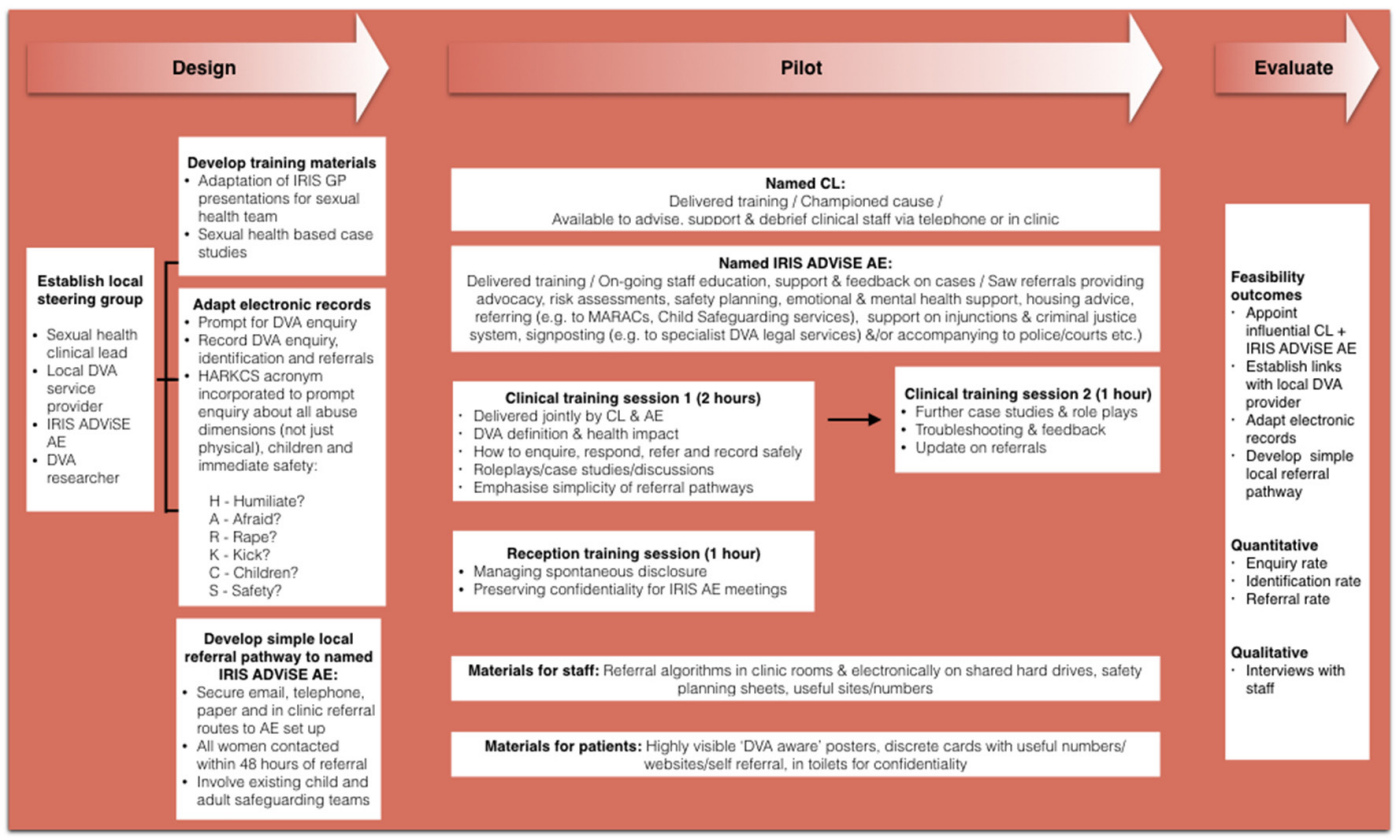
IRIS ADViSE adaptive pilot study: core methodology.

Figure 1 summarises the core methodology including the IRIS ADViSE intervention details. Sexual health-based case studies integrating best practice in the healthcare response to DVA with routine sexual healthcare were included. Local steering groups facilitated discussion on how best to develop and implement IRIS ADViSE locally. At site 1, adaptation of electronic records and paper triage forms occurred simultaneously; while at site 2, a question on abuse was added to the paper triage form, 3 months prior to the abuse questions’ integration into the electronic records. Patients on arrival at reception complete the paper triage form that contains questions about reason for attendance.

Table 1 summarises how site 1 informed implementation at site 2. At site 1, it was not mandatory for staff to indicate whether they had asked about DVA whereas it was at site 2. This difference was instituted directly due to the results from site 1.

**Table 1:** IRIS ADViSE adaptive pilot study: how site 1 informed site 2?

Site 1	How site 1 informed site 2?	Site 2
Clinical training sessions: Two open to staff attending on the day.	Informal feedback to AE and qualitative interview revealed staff that had had no IRIS training. Tried to improve access to training for all clinicians working in the service.	Additional abridged training sessions held for those unable to attend the main training sessions.
Electronic records’ adaptation: Six HARKCS questions on DVA, within a template, inserted into pro forma. Each question required a ‘yes’ or ‘no’ answer, alongside free text boxes: H—Humiliate? A—Afraid? R—Rape? K—Kick? C—Children? S—Safety?	Electronic quantitative results showed that HARKCS template regularly completed incorrectly, for example, queries about children and safety made, even when no DVA identified. Potential for clinicians to just read out questions in a tick box manner. Reflective discussion at site 2 led to decision to use HARKCS image, reminding clinicians to ask about the multiple dimensions of DVA, including emotional, sexual, physical abuse and coercive control, related to being afraid. Fewer initial questions Prompts to enquire about children and safety only appearing if there was a DVA disclosure.	HARKCS image added to pro forma. Two DVA questions inserted into pro forma: 1. Have you asked about DVA? Y/N 2. Has DVA been disclosed? Y/N If DVA disclosed, two more questions appear: 1. Immediate safety at risk? 2. Patient has children? With free text box for recording of referral details.
HARKCS questions did not have to be filled in but could be skipped.	Site 1: Enquiry rate 10%, identification rate 4%, referral rate 50%. Tried to improve these rates by making it mandatory to indicate whether DVA had been asked about.	Mandatory for staff to indicate whether they had asked about DVA (‘yes’ or ‘no’) before they could complete the electronic notes. Site 2: Enquiry rate 61%, identification rate 7%, referral rate 10%.
Evaluation: Pretraining and post-training sessions’ questionnaires used—low rates of return; given out to staff not delivering care at female wal in service—inappropriate to evaluate.	Tried to improve return of questionnaires—their completion aligned closely to receiving a certificate of continuing professional development (CPD) and attendance.	Pretraining and post-training sessions’ questionnaires when completed exchanged for a certificate confirming attendance at CPD session. Evaluation showed average self-rated knowledge on DVA health consequences, enquiry, response and how to make advocacy referrals increased by 40%.
Four qualitative interviews, with staff; initial pilot interview carried out by academic GP. Other interviews by independent qualitative researcher.	Topic guide for qualitative interview first constructed by academic GP. Revised and improved by independent qualitative researchers at site 1 and later at site 2, where it was used for a more comprehensive qualitative study. Results published separately.	17 qualitative interviews by independent qualitative researcher.

AE, advocate-educator; DVA, domestic violence and abuse; GP, general practitioner; IRIR ADViSE, Identification and Referral to Improve Safety while Assessing Domestic Violence in Sexual Health Environments; HARKCS, template questions for asking about domestic violence and abuse—please see details in second row of table.

### Ethical aspects

We sought the views of chairs of local ethics committees at both sites. We received written confirmation that the collection of data from the medical record constituted routine service evaluation.

### Outcome measures and data collection

The predefined feasibility outcome measures were whether

- a DVA clinical lead was appointed in each sexual health service
- a local DVA services’ provider collaborated with the sexual health service on this project
- an advocate educator was appointed
- an electronic *pro forma* included assessment of DVA
- local referral pathways were developed
- enquiry, identification and referral rates were measurable.

Handwritten notes were kept on the progress of the project by AS at site 1 and NP at site 2. Anonymised aggregated data, following the first session of training, once the pilot project was live was collected for 7 weeks at site 1 and 12 weeks at site 2, on the number of women who

- attended the walk-in sexual health service at east London and Bristol
- were asked about DVA by staff
- were identified as being affected by DVA, either currently or historically
- were referred to the AE.

We aimed to collect 3 months of baseline data at each site.

## Results

At both sites, all feasibility outcomes were achieved, in that process measures (appointing key staff; see below) were attained and potential future outcome measures (enquiry, identification and referral rates) were measurable.

### Process measures attained

Local sexual health consultants were appointed as DVA CLs, local DVA service providers collaborated on the project and AEs, experienced in advocacy and training, were successfully recruited. DVA enquiry was integrated into the standard electronic template, reminding sexual health staff to ask about DVA. Adapted IRIS training was delivered to the sexual health team in their weekly continuing professional development education session. Clear local referral pathways on how best to contact the IRIS AE were established.

### Outcome measures measurable

At site 1, over 7 weeks, the DVA enquiry rate was 10% with 267 women out of 2568 women attending asked about DVA. The DVA identification rate was 4% with 12 out of the 267 women asked about abuse, affected by abuse. Four additional cases of DVA were identified via the self-triage form. Overall 50% (8 out of 16) of the women affected by abuse were referred to the AE.

At site 2, over 12 weeks, the DVA enquiry rate was 61% with 1090 women out of 1775 women attending asked about DVA. The DVA identification rate was 7% with 79 out of the 1090 women asked about abuse affected by abuse. At site 2, 13 of the 79 cases of DVA were identified via the self-triage form. Overall 10% (8 out of 79) of the women affected by abuse were referred to the AE.

During the 3 months preceding the start of each pilot project, there were no electronically recorded cases of DVA identified and no referrals to DVA specialist services. At site 2, during this 3-month period, it is unknown how many women disclosed abuse on the paper self-triage form but none were referred to specialist services.

## Discussion

This adaptive pilot study shows that it is feasible to develop and implement an IRIS-based DVA training and referral package for sexual health clinics. The intervention resulted in the identification and referral of women affected by DVA, suggesting that it is a potentially effective intervention. It is also feasible to collect data for quantitative evaluation of the intervention’s impact on DVA enquiry, identification and referral for advocacy.

The actual rates of DVA enquiry, identification and referral, all show a change from baseline which means that IRIS ADViSE affects clinician behaviour. The limitations of this study is that it is not powered to estimate the size of this effect, nor can it conclusively exclude a secular trend due to the non-randomised study design.

This study’s strength was its adaptive design, allowing transparent, sequential, rapid refinement of IRIS ADViSE. Piloting IRIS ADViSE in more than one site acknowledges variation in sexual health service delivery and confirms the feasibility of tailoring the intervention to individual services.

Comparison of sites 1 and 2 shows that the DVA enquiry rate increased over fivefold, from 10% to 61% with just under a doubling in the DVA identification from 4% to 7% and a drop in referral rates from 50% at site 1% to 10% at site 2, meaning that the absolute numbers of women referred to an AE at the two sites was the same (eight women at each site). These differences are most likely to be due to the adaptive change made at site 2, making it mandatory for clinicians to indicate whether DVA had been asked about before proceeding.

As IRIS ADViSE is introduced into diverse sexual health services and settings, the local steering groups should consider the cons and pros to each approach. For example, making it mandatory to record whether DVA enquiry has occurred appears to be associated with a drop in the referral rates through higher enquiry and identification rates. IRIS ADViSE may benefit women affected by DVA who are asked about DVA but decide to not disclose (large numbers as estimated by DVA survey prevalence figures) and may benefit women identified as being affected by DVA who are offered referral but decide that they do not want to be referred.

A recent DVA screening study, in multiple hospital settings, found that in genitourinary medicine 5.7% patients screened reported ever experiencing DVA, using a training intervention delivered by a local specialist DVA provider,^20^ without clinician-delivered clinically relevant training, nor using validated DVA questions like HARK,^21^ nor reporting how abuse questions were integrated into the medical record. This intervention’s DVA enquiry rate (ie, the number of patients asked about DVA) and referral rate for advocacy are unknown (ie, source of referrals received by the hospital-based domestic violence advisor undetermined). To allow a quantitative comparison with IRIS ADViSE requires the latter’s identification rates to be adjusted so that the denominator is changed from the total number asked about DVA to the total number who attended the service. At site 1, this adjusted identification rate is 0.6% (= 16/2568); at site 2 this is 4.5% (= 79/1775), which is a similar level to the Warren-Gash *et al* study. These figures support and we recommend retaining site 2’s mandatory recording of whether DVA enquiry has occurred.

In summary, the IRIS intervention has been successfully adapted for female walk-in sexual health services. Further evaluation of IRIS ADViSE with appropriate refinement for each setting is now required to confirm its effectiveness prior to national scaling up. A quasi-experimental approach would enable a pragmatic phased implementation of IRIS ADViSE to other sexual health clinics, including HIV clinics, pregnancy advisory services, outreach services, psychosexual clinics, male and LGBT services. Future studies, using this adaptive, novel approach, should extend DVA enquiry ensuring a competent and compassionate clinical response whenever abuse is identified.
